# Medical-Grade PLA Nanocomposites with Optimized Tungsten Carbide Nanofiller Content in MEX Additive Manufacturing: A Rheological, Morphological, and Thermomechanical Evaluation

**DOI:** 10.3390/polym15193883

**Published:** 2023-09-25

**Authors:** Nectarios Vidakis, Amalia Moutsopoulou, Markos Petousis, Nikolaos Michailidis, Chrysa Charou, Nikolaos Mountakis, Apostolos Argyros, Vassilis Papadakis, Evgenia Dimitriou

**Affiliations:** 1 Department of Mechanical Engineering, Hellenic Mediterranean University, 71410 Heraklion, Greece; amalia@hmu.gr (A.M.); markospetousis@hmu.gr (M.P.); charou@hmu.gr (C.C.); mountakis@hmu.gr (N.M.); 2 Physical Metallurgy Laboratory, Mechanical Engineering Department, School of Engineering, Aristotle University of Thessaloniki, 54124 Thessaloniki, Greece; nmichail@auth.gr (N.M.); aargyros@auth.gr (A.A.); evgeniaod@auth.gr (E.D.); 3 Centre for Research & Development of Advanced Materials (CERDAM), Center for Interdisciplinary Research and Innovation, Balkan Centre, Building B’, 10th km Thessaloniki-Thermi Road, 57001 Thessaloniki, Greece; 4 Department of Industrial Design and Production Engineering, University of West Attica, 12244 Athens, Greece; v.papadakis@uniwa.gr; 5 Institute of Electronic Structure and Laser of the Foundation for Research and Technology-Hellas (IESL-FORTH)—Hellas, N. Plastira 100m, 70013 Heraklion, Greece

**Keywords:** additive manufacturing (AM), material extrusion (MEX), mechanical properties, polylactic acid (PLA), tungsten carbide (WC), hybrid polymer/ceramic

## Abstract

The goal of this paper is to investigate tungsten carbide (WC) as a reinforcement in the popular material extrusion (MEX) additive manufacturing (AM) procedure. The impressive characteristics of WC demonstrate its potential as a valuable additive for commonly used polymeric matrices in MEX 3D printing, offering reinforcement and stabilization properties. The mechanical properties of hybrid polymer/ceramic nanocomposites made up of various filler loadings (0–10 wt. %) of medical-grade polylactic acid (PLA) and WC were studied. The mechanical characteristics, structure, and thermomechanical properties of the resulting compounds were fully characterized following the respective standards. The fracture mechanisms were revealed with Scanning Electron Microscopy. Overall, a laborious effort was implemented with fifteen different tests to fully characterize the nanocomposites prepared. In comparison to the raw PLA material, the tensile strength of the 4.0 wt. % WC PLA/WC nanocomposite was improved by 42.5% and the flexural strength by 41.9%. In the microhardness test, a 120.4% improvement was achieved, justifying the properties of WC ceramic. According to these findings, PLA nanocomposites reach high-performance polymer specifications, expanding their potential use, especially in wear-related applications.

## 1. Introduction

Over the past decade, additive manufacturing has achieved significant advances that have led to its extensive use in a variety of fields, such as the manufacturing of engine parts, replacement components, as well as food, and organic parts [1,2]. Due to the extensive use of additive manufacturing, there is a rising need for materials that offer durability, remarkable mechanical characteristics, and other favorable features in 3D-printed objects [3,4,5].

FFF (Fused filament fabrication), which is a material extrusion (MEX) method, is recognized as the most widely used technique for the additive manufacturing of thermoplastic materials, among the different additive manufacturing techniques [6,7,8]. By melting the raw material into a molten polymer thread, FFF constructs parts gradually by laying consecutive layers [9]. This fabrication method uses filaments made of various polymers, such as polylactic acid (PLA) [10,11], acrylonitrile butadiene styrene (ABS) [12], polyethylene (PE), polypropylene (PP), and other related substances [13]. Additionally, the employment of metal–polymer [14] and ceramic–polymer [12,15,16] composites has increased recently. These composite materials present distinct properties, such as extreme hardness and electrical conductivity, that cannot be achieved by the 3D printing of pure polymers [17,18,19,20]. Additionally, it has been discovered that the addition of small quantities of ceramic powders, such as oxides, carbides, nitrides, or borides, can significantly amend the properties of the 3D-printed parts. These additives result in a notable increase in strength in addition to a noticeable reduction in the rate of wear of the final manufactured parts [20,21].

The biodegradable polylactic acid (PLA) thermoplastic is frequently utilized in many different applications, including 3D printing, due to its remarkable mechanical qualities [22,23]. PLA is valued for its biocompatibility, low toxicity, and appropriateness for tasks requiring low mechanical stress [24,25]. In addition to its mechanical response in MEX 3D printing [26,27], the impact of the 3D-printing factors on the quality characteristics of the PLA polymer has been reported [28,29]. The applicability of PLA as a matrix material in compounds has been conclusively proven by extensive investigations in MEX 3D printing [30,31]. Composites have been made using a variety of fillers, such as glass fiber [32], or carbon black [33]. Additionally, the qualities of the matrix material have been improved while also maintaining environmental friendliness [34] by combining PLA with wood [35]. To improve the shape accuracy and surface roughness of the nanocomposites post-processing with CO_2_, the laser cutting of MEX parts was implemented, which made them appropriate for cutting-edge cases that require nanocomposites with high-quality characteristics related to dimensional accuracy [30]. Due to its biocompatibility and eco-friendliness, PLA has been investigated for use in biomedical applications [36,37]. To maintain the eco-friendliness of the polymer, organic additives have been investigated for their reinforcing performance [38]. Its sustainability has been investigated through the optimization of its energy consumption during the MEX 3D-printing process [26] and its performance during recycling [39].

Because of their advanced and adaptable qualities, ceramics are widely used at present in a variety of sectors [40]. Carbides, which are ceramic materials, exhibit remarkable mechanical characteristics, particularly in terms of wear resistance. As a result, they find applications in coatings and cutting tools due to their exceptional properties [41], or gear hobbing applications [42]; both applications are indicative of the capabilities of these materials. New needs in the industry, such as the mechatronics, chemical, and biomedical sectors, have found valuable applications for advanced ceramics with outstanding functions and unique geometric properties [43]. Unlike conventional manufacturing processes, additive manufacturing (AM) permits the creation of intricately shaped ceramic structures while also imparting high durability, thermal stability, and chemical resistance [4,44]. As a conventional method for ceramic additive manufacturing (AM), the utilization of the fused filament fabrication process of polymeric filaments containing ceramic particles was used [45]. Another study conducted by this team explored the use of two biocompatible materials to increase mechanical properties by combining a pure PLA matrix with titanium nitride (TiN) particles as a filler. TiN nanoparticles were discovered to enhance PLA’s mechanical properties when used in MEX 3D printing. According to the findings, a nanocomposite with a 4.0 wt. % of TiN load improved the tensile strength by 43.4% and the flexural strength by 51.5% [15].

Because of its exceptional characteristics, including high hardness, low density, great chemical stability, and performance at elevated temperatures, tungsten carbide (WC) has been established as a ceramic material with the most potential [46]. The anti-abrasion and mechanical qualities of composite materials are frequently improved by the addition of tungsten carbide (WC) particles in the form of powder as an additive. In their research, Visconti et al. added silicon powder and WC powder to a glass/epoxy compound. When WC powder was included in the matrix material, the findings revealed a substantial improvement in the wear resistance, especially under harsh wear conditions [47]. Additionally, Mohan et al. investigated the effects of adding WC powder to glass/epoxy compounds and found that the composites had an improved tribological performance [48]. WC’s unique properties have been exploited for advanced medical applications and devices [49], such as in radiation shielding [50]. In 3D printing, WC has been investigated in Binder Jet 3D Printing (BJ3DP) and Selective Laser Sintering (SLS) for metal parts’ production since it is a hard-to-process metal [51,52], mainly for applications requiring high wear resistance [53,54]. In FFF AM technology, research is still limited. WC has been investigated as a reinforcement for the acrylonitrile butadiene styrene (ABS) polymer [55].

The goal of this investigation is to produce nanocomposites for the MEX 3D-printing technique with improved mechanical properties. In this context, the effect of tungsten carbide nanoparticles as a filler for a medical-grade PLA polymer is evaluated. The addition of a ceramic filler in a polymeric matrix can affect various aspects of the material in addition to the mechanical properties, such as the thermal properties, its processability, and its morphological characteristics, when 3D-printed. For completeness and to fully characterize the prepared nanocomposites, a range of thermomechanical examinations was conducted on the fabricated filaments and the 3D-printed specimens. The ASTM requirements were followed during these investigations to have comparable results with other materials. The goal of this investigation is to comprehend how changes in tungsten carbide particle loadings affect the form and surface properties of the produced filament samples. The promising results of this study broaden the spectrum of potential materials and applications while highlighting the potential of PLA nanocomposites in MEX 3D printing.

## 2. Materials and Methods

**Figure 1:**
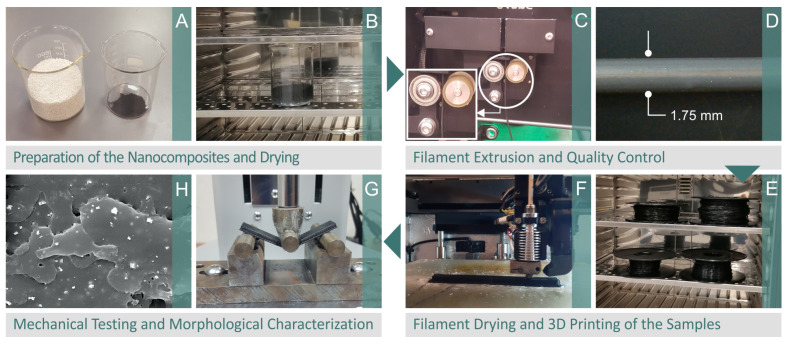
Flow diagram illustrating the experimental approach used accompanied by photos that show each step of the process (**A**) raw material, (**B**) drying of the raw material, (**C**) filament extrusion, (**D**) filament inspection, (**E**) filament drying, (**F**) MEX 3D printing of the samples, (**G**) mechanical testing (three-point bending is presented in the image), (**H**) morphological analysis with SEM.

Figure 1 displays the investigational method employed to fabricate the samples and the analysis of their thermomechanical and morphological characteristics. Pictures of the pure materials throughout the 24-h drying procedure at 60 °C are shown in Figure 1A,B. The filament extrusion process is displayed in Figure 1C, coupled with the evaluation of the filament diameter (Figure 1D). The filament produced in the lab was maintained in a dry atmosphere for 24 h at 60 °C to eliminate any remaining moisture (Figure 1E). The additive manufacturing procedure adopted to produce the test samples is demonstrated in Figure 1F. Finally, Figure 1G,H depict the 3D-printed specimens that were subjected to mechanical and structural investigations.

### 2.1. Materials

The samples were produced using a coarse powder of medical-grade PLA (known as Y-F PLA), which served as the matrix material. The manufacturer’s information detailed the specifications of this medical-grade PLA as follows: tensile strength of ~50 MPa, melting point of ~160 °C, MFR = 1–30 (190 °C, 2.16 kg), and density of 1.25 g/cm^3^. Nanographi in Ankara, Turkey, provided the tungsten carbide (WC) nanopowder, which has a purity of 99.9%, a specific surface area of 1.5–2.0 m^2^/g, a melting point of 2870 °C, a particle size of 150–200 nm, true density of 15.7 g/cm^3^, and hexagonal crystal phase. To produce the above-mentioned nanocomposites, the medical-grade PLA polymer and the tungsten carbide powders were combined. This filler was used to increase the nanocomposites’ overall quality and properties in comparison to the pure PLA matrix. No other additives or plasticizers were added to the nanocomposites and, thus, the clear effect of WC in the PLA matrix was derived from the experimental procedures.

### 2.2. Development of Nanocompounds

**Figure 2:**
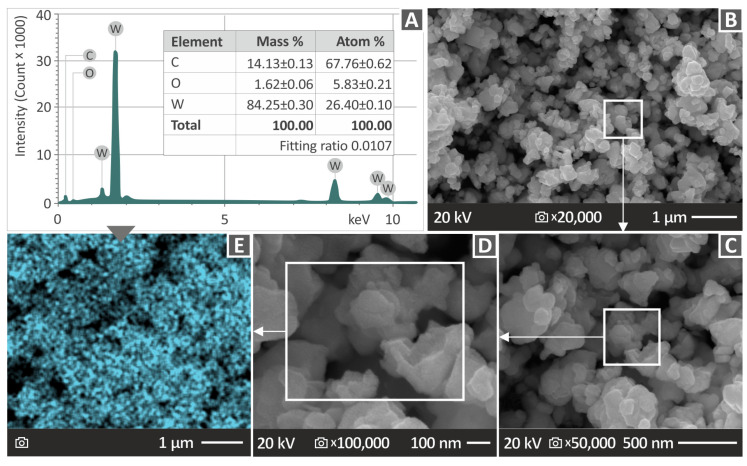
The investigation of the WC powder. (**A**) EDS graph analysis to determine the elemental composition, (**B**) SEM images captured at 20,000× amplification, (**C**) SEM images captured at 50,000× amplification on the region shown in (**B**), (**D**) SEM images captured at 100,000× amplification on the area presented in (**C**), and (**E**) EDS mapping on the region shown in (**D**) demonstrating the uniform dispersion of the tungsten (W) element.

The WC powder used in this study was initially examined using SEM (Scanning Electron Microscopy) to evaluate its morphological features before creating the compounds. The JSM-IT700HR, which is a field-emission SEM device made by Tokyo, Japan-based Jeol Ltd., was the specific instrument used for this evaluation. EDS measurements were also executed using the same instrument to ascertain the chemical components, as displayed in Figure 2. The identification of the carbon (C) and tungsten (W) quantities, which showed the most distinct peaks in the EDS analysis, is shown in Figure 2C. Additionally, oxygen was detected, constituting approximately 1.62% of the mass, which could potentially be attributed to moisture in the WC particles. The EDS graph verified that the chemical composition of the WC particles is consistent with the manufacturer’s specifications.

The SEM images were examined to confirm the shape and size of the WC particles, as demonstrated in Figure 2D,E. The results of the EDS mapping for the tungsten (W) component of the WC particles are shown in Figure 2E The mapping shows that W is mostly equally distributed, with just a few voids or small, isolated regions showing fluctuations in the W concentration.

As previously mentioned, the raw materials were heated at a 60 °C temperature for 24 h prior to the creation of the nanocomposites in order to remove any remaining moisture. Five different material blends were created by varying the weight percentage (wt. %) of WC with concentrations ranging from 0.0 to 10.0 wt. %. At room temperature (23 °C) and 4000 rpm for 30 min, a high-wattage blender was used to achieve the first distribution of the WC particles in the PLA polymer, with the virgin materials still in powder form. The combinations underwent an additional drying step after the blending phase (60 °C, 4 h). Filaments were created with these powder blends by using a Noztek extruder from Shoreham-by-Sea, England. This step of the process aimed to distribute the WC particles in the PLA matrix through a thermomechanical extrusion process and fabricate nanocomposites in filament form. Then, a 3devo shredder from Utrecht, Netherlands, was used to shred the created filament into pellets. The pellets went through processing, and the final filaments were produced, utilizing a 3devo Composer from Utrecht, Netherlands. The 3devo Composer extruder used in this work incorporates a screw configuration that is specially made for blending and melting materials in order to produce filaments suitable for MEX 3D printing. This second extrusion process aimed to improve the WC distribution in the PLA matrix and produce the final nanocomposites in filament form with a uniform and the best possible filler distribution in the matrix, with the following methodology. The heating temperature on the 3devo Composer extruder’s first and fourth heating zones was 170 °C and that of the second and third heating zones was 190 °C. To enable adequate filament cooling throughout the production process, the cooling fan speed was set to 55%, while the rotational speed of the extrusion screw was set at 5 rpm. As a result, of the above-mentioned extrusion parameters, a filament with a consistent 1.75 mm diameter was successfully manufactured. Filament diameter deviation was acceptable for the MEX 3D-printing method.

As stated, to enhance the distribution of the WC nanoparticles within the polymer matrix, the two previously mentioned extrusion procedures were used. As stated above, no further additives or compatibilizers were used during the construction process of the nanocomposites. The same process was used to extrude the filament of the pure PLA material, which was used as a standard against which the mechanical responses of the fabricated nanocomposite materials were examined.

### 2.3. Three-Dimensionally Printed Samples’ Production Process

**Figure 3:**
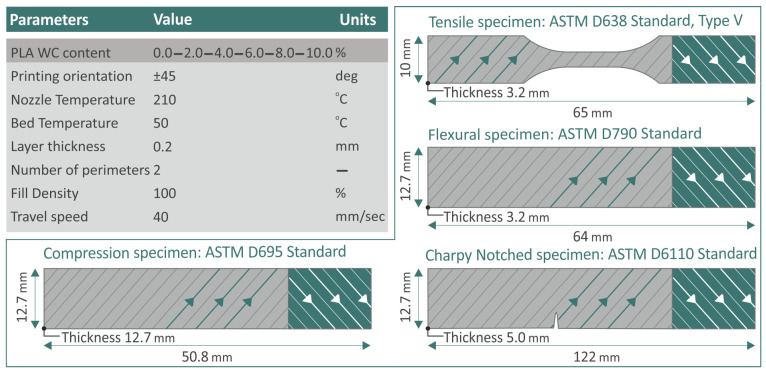
The 3D-printing factors utilized for specimen fabrication, alongside the ATSM standards for each experiment.

Two extrusion steps were employed to create filaments made of PLA/WC nanocomposites at the aforementioned loadings. The filament of the pure polymer was also created as the control sample. These filaments were utilized to create specimens using the Intamsys Funmat-HT 3D Printer from Shanghai, China. To identify the factors for generating 3D-printed objects, the software platform Intamsuite (version 4.2), also created by Intamsys (Shanghai, China), was utilized, and these parameters were established through a process of trial and error before starting the sample manufacturing procedure and by referring to the corresponding literature, for example, [15]. Then, the required G-codes for the 3D-printing procedure were produced. For each test, the samples were produced in compliance with the dimensional requirements listed in the relevant ASTM specifications. These specifications cover the following: ASTM D638-02a [56] for conducting tensile experiments, ASTM D790-10 [57] for conducting flexural investigations, ASTM D6110-02 [58] for conducting Charpy notched impact investigations, and ASTM D695 [59] for conducting compression tests. The samples were produced with a 100% infill, indicating that the interior of the 3D-printed object was almost solid [15]. The rectilinear infill pattern, which fills the object’s interior space with a grid-like structure, was used. In addition, the raster direction was altered from +45 degrees to −45 degrees for each subsequent layer. A summary of the 3D-printing factors utilized in this investigation is shown in Figure 3.

### 2.4. Analysis of Thermal Properties and Rheological Behavior

The thermal properties of the composites were examined using the filament samples. Thermogravimetric analysis (TGA) experiments were conducted to assess the compounds’ capacity to preserve their structural formation at elevated temperatures. The measurements were conducted in N_2_ as an inert atmosphere with a PerkinElmer Diamond apparatus by PerkinElmer, Inc. (Waltham, MA, USA). Starting at ambient temperature and with a rising rate of 10.0 °C/min, the temperature gradually increased until it reached 550 °C. Furthermore, DSC analysis was conducted to examine the nanocompounds’ thermal characteristics. A TA Instruments Discovery Series DSC-25 by TA Instruments (from New Castle, DE, USA) was used with a temperature cycle that was in the range of 25–300 °C and a heating rate of 15 °C per minute for these measurements. No heating/cooling cycles were applied prior to the measurements.

A DHR-20 Discovery Hybrid Rotational Rheometer by TA Instruments (from New Castle, DE, USA) featuring an Environmental Test Chamber with an accurate regulation of the temperature and two parallel plates was utilized to assess the rheological parameters using filament melts. With the use of this rotational rheometer, it was possible to monitor how a liquid sample’s shape changed in reaction to an external force. To ensure the substance’s fluidity and avoid any consequences connected to decomposition, experiments were conducted above the melting point of the substance and below the decomposition temperature. To avoid sample damage and overheating, each data point was recorded for 10 s. Melt flow rate (MFR) measurements were conducted to evaluate the polymeric material’s flow properties under particular temperature and pressure settings. For these measurements, the material had to be sent through a hole with a defined length and diameter. The approach followed ASTM D1238-13 [60], a global standard that outlines the melt flow rate (MFR) testing process.

### 2.5. Analysis of Raman Spectroscopy

A specially designed Raman spectrometer LabRAM HR (from HORIBA Scientific, Kyoto, Japan), was used to conduct the Raman analysis. A module for a solid-state laser with a supreme output power of 90 mW and a central operating wavelength of 532 nm was used as the excitation source for Raman spectroscopy. The samples were illuminated with excitation light, and Raman signals were collected using a microscope (LMPlanFL N, Olympus, Tokyo, Japan) with a 10.6 mm operating distance, a 0.5 numerical leak, and a 50 microscopic objective lens. To lower the power on the specimen to 2 mW, a 5% transmittance neutral density filter was also employed. The laser point size, as a result, was roughly 2 μm axially and 1.7 μm laterally. Each measurement took 50 s to acquire, with five accumulations being conducted at each point. Three optical windows were provided by the acquired Raman spectrum ranging from 50 to 3900 cm^−1^, and the resolution of the Raman spectrum was about 2 cm^−1^. The spectrometer’s 600-groove grating was used.

### 2.6. Evaluation of the Produced Filaments

Prior to the 3D-printing method to create the specimens, all the generated filaments underwent surface examination and measurements of their diameter and tensile strength. To ensure compliance with the requirements, a closed-loop control system continuously monitored the filament’s diameter (a built-in feature of the 3devo Composer extruder). To verify the diameter measurements, a digital caliper was also used. Imada Inc., Northbrook, IL, USA’s Imada MX2 apparatus was used to measure the filament’s tensile strength. The experiments ran at 10 mm/min speed. For every compound, five specimens in total were analyzed.

To analyze the surface morphology of each nanocomposite, AFM was utilized. The quantifications were accomplished in ambient air employing an XE7 AFM system (from Park Systems, Seoul, Republic of Korea). Photos were obtained employing a Nanosensors NCHR cantilever from the USA, working at a frequency of approximately 300 kHz and fitted with a tip diameter of 10 nm. The images were captured using the intermittent contact technique at a 0.5 Hz scanning rate on a 10 μm × 10 μm area of the lateral surface of the filaments. During imaging, a constant working set point with an amplitude exceeding 70% of the natural oscillation was retained to capture pictures without the influence of external forces.

### 2.7. Assessment of the Mechanical Properties

A series of tests were performed following the ASTM protocols to assess the thermomechanical characteristics of the 3D-printed objects under several loads or external forces. These tests were accomplished to estimate the material’s overall strength, stiffness, resistance to fracture, and capacity for deformation under applied loading. The tests also aimed to determine how WC nanoparticles affected the mechanical performance of the nanocompounds. Standard ambient conditions of 23 °C and relative humidity of 55% were kept during the investigations to avoid environmental factors. Additionally, for repeatability and comparison reasons, all of the experiments conducted used the same set of test parameters.

For the analysis of the PLA/WC nanocompounds, five samples were produced and examined for all the created composites. Tensile examinations were executed using an Imada MX2 apparatus (from Imada Inc., Northbrook, IL, USA), at a 10 mm/min strain rate using standard grips. With a 1.3 mm/min testing rate, compression investigations were conducted utilizing Instron KN1200 equipment from Norwood, MA, USA. Flexural investigations were conducted utilizing Imada Inc’s MX2 equipment (from Northbrook, IL, USA) with a 52.0 mm support span and a 10 mm/min strain rate. Charpy notched samples were used in the impact examinations, which were conducted using Terco MT220 equipment (from Kungens Kurva, Sweden) and a 367 mm hammer release height. Using InnovaTest-300 equipment (from Maastricht, The Netherlands), a Vickers hardness test was conducted. A 200 gF stress was applied for 10 s to acquire the microhardness measurements.

### 2.8. Examination of the Structure and Morphology of the 3D-Printed Specimens

The form and structure of the shattered and lateral surfaces of the tensile test 3D-printed parts were carefully investigated by employing a JSM-IT700HR field emission scanning electron microscope by Jeol Ltd. (from Tokyo, Japan). The microscope used an acceleration voltage of 20 kV while operating in a high vacuum. SEM was also used to take pictures of the gold-sputtered specimens at several magnifications, enabling a thorough inspection of the samples’ surfaces. EDS and EDS mapping were also performed to verify the presence of the NPs in the fracture surfaces (internal structure of the parts) and to locate possible agglomerations of the NPs in the nanocompounds.

## 3. Results

### 3.1. Estimation of Thermal Characteristics through Thermogravimetric Analysis and Differential Scanning Calorimetry

**Figure 4:**
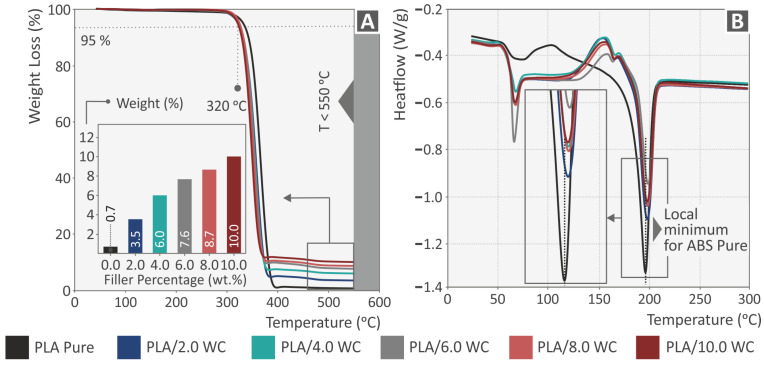
Thermal characterization of the raw PLA and PLA/WC nanocompounds using (**A**) TGA curves and (**B**) heat-flow curves at different temperature ranges.

The weight loss of the studied composites and pure PLA matrix is shown in relation to the temperature in the TGA graphs depicted in Figure 4A. It is evident from the data presented in Figure 4A that the PLA material’s resistance to thermal degradation is not considerably affected by the addition of WC nanoparticles. The weight loss started approximately at 320 °C for all the filled samples, which is commensurate with the temperature at which the pure PLA begins to lose weight. As demonstrated, the residual weight percentage in the samples is close to the filler percentage. It is important to note that the temperatures used in extrusion procedures, such as filament manufacture and MEX 3D printing, were lower than the temperature where the weight loss began.

Figure 4B displays the heat flow curves of the produced nanocomposites as well as the provided PLA matrix. Table 1 presents the glass transition (T_g_), crystallization (T_c_), and melting (T_m_) temperatures and the crystallization (ΔH_c_) and melting (ΔH_m_) enthalpies, as well as the (%) crystallinity (X_c_) of the composite filaments. X_c_ (%) was calculated using Equation (1), where φ is the percentage of the PLA matrix in the composite and ΔH_0_ is the melting enthalpy of the 100% perfectly crystalline PLA (93 J/g) [61,62]:

(1) $$X_{c}\left(\%\right)=\frac{{\Delta H}_{m}+{\Delta H}_{\mathrm{cc}}}{{\Delta H}_{0}\varphi }\cdot 100$$

The melting and crystallization enthalpies were calculated by integrating the area under/over the respective transition of the DSC graph.

The behavior of both the composite and pure materials is the same for the glass transition (around 60 °C) and the melting temperatures (around 200 °C) and agrees with the literature [63]. However, with respect to the crystallization temperature, the filled samples exhibited higher crystallization temperatures and enthalpies. As the filler percentage increases, the percentage of PLA decreases, leading to a decrease in both enthalpies’ absolute values. By contrast, the crystallinity increases as the filler particles act as crystal nucleation sites, forming ordered crystal structures in the polymer matrix [64]. This relationship between filler content and crystallinity has previously been reported in the literature [65,66,67]. The double crystallization peak in the filled samples can be attributed to the presence of two crystal lamella types of different thicknesses. The peak appearing at the lower temperature corresponds to the thinner lamellae, whilst that appearing at the higher temperature corresponds to the thicker lamellae [68]. Alternatively, the first peak may derive from the lamellae initially present in the material, while the partially melted amorphous material may recrystallize into more structured lamellae, requiring higher temperatures to melt [69,70].

### 3.2. Examination of the Rheological Properties

**Figure 5:**
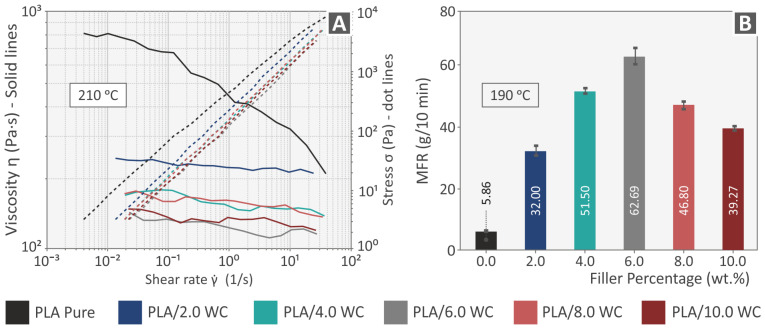
Rheological investigation of the pure PLA polymer and PLA/WC composites: (**A**) viscosity and stress versus shear rate and (**B**) melt flow rate versus filler percentage.

Figure 5A illustrates the findings of the rheological analysis performed at 210 °C, including the viscosity and stress as functions of the shear rate depicted on the logarithmic axes. It is worth mentioning that, although the DSC curves revealed that the filament had not melted completely at 210 °C, the temperature for the viscosity measurements was set at 210 °C to best represent the printing temperature. The temperature of 210 °C ensured the appropriate viscosity for the printing of the filaments; lower temperatures would result in the clogging of the printhead, whilst higher temperatures would cause the filament to flow uncontrollably from the printing unit. The experiments show that the viscosity generally decreased with the rise of the induced shear rate for all the composites, indicating a non-Newtonian, shear-thinning, or pseudoplastic behavior. This behavior is desirable for materials that are intended to be used with 3D printing because it allows for a smooth flow from the nozzle to the print bed and the retention of the extruded shape [71]. Contrary to the typical viscosity increase with the increase in the nanoparticle content, the viscosity values of the examined composites were lower in comparison with the pure material in the shear rate region examined. The incorporation of the hard ceramic filler in the polymer matrix resulted in greater values of particle packing, which is something that leads to particle–particle interactions rather than particle–polymer interactions and can explain the latter behavior of the composites [72]. Additionally, due to the filler particles’ slippage phenomenon, a reduction in the composite material’s chain strength against the applied shear stresses was observed when conducting the rheological experiments [73,74]. As the concentration of WC nanoparticles increases within the PLA matrix, there is a higher chance of particle–particle interactions. Consequently, these interactions can promote particle rearrangement and slippage, allowing nanoparticles to slide past each other more easily under the influence of shear stress. In contrast to the traditional scenario where nanoparticles act as obstacles, impeding the movement of the polymer chains and leading to increased viscosity, the particle–particle interactions, and subsequent slippage in the PLA-WC composite facilitate the enhanced mobility of the nanoparticles. This increased mobility can reduce the overall resistance to shear flow within the material. Another contributing factor to the viscosity reduction is the change in particle packing efficiency as the nanoparticle concentration increases. With higher levels of WC nanoparticles, there is a greater potential for close packing due to the increased particle density. This enhanced particle packing might lead to an increased probability of particle–particle contact and slippage, which in turn can reduce the effective viscosity of the composite material. Particle roughness can still play a role in facilitating slippage within the polymer–nanoparticle composite. The roughness of nanoparticles introduces microscale irregularities on their surfaces, creating localized regions of reduced contact with the polymer matrix. This phenomenon can result in several effects that promote easier particle movement and reduced viscosity. For example, the irregularities on the particle surfaces can disrupt the close contact and strong adhesion between the nanoparticles and the surrounding polymer matrix. As a result, the cohesive forces between the nanoparticles and the matrix are weakened. This weakened interaction allows for easier particle–polymer separation and mobility, contributing to slippage. Another potential result is that the rough regions on particle surfaces may act as micro-lubricating sites. When subjected to shear forces, the polymer chains in the matrix could penetrate these rough regions, forming lubricating layers that reduce the friction between the particles and the matrix. This lubrication effect promotes easier particle movement and slippage.

Figure 5B shows the melt flow rate (in g/10 min), measured at 190 °C, as a function of the filler weight percentage. The material flow rate increased with the addition of the particles in comparison to the pure PLA, which is also consistent with the rheometry experiments described previously. The greatest flow rate value was observed at a filler loading of 6 wt. %, which is consistent with the lowest viscosity value obtained at the same filler loading.

### 3.3. Raman Spectroscopy Examination

**Figure 6:**
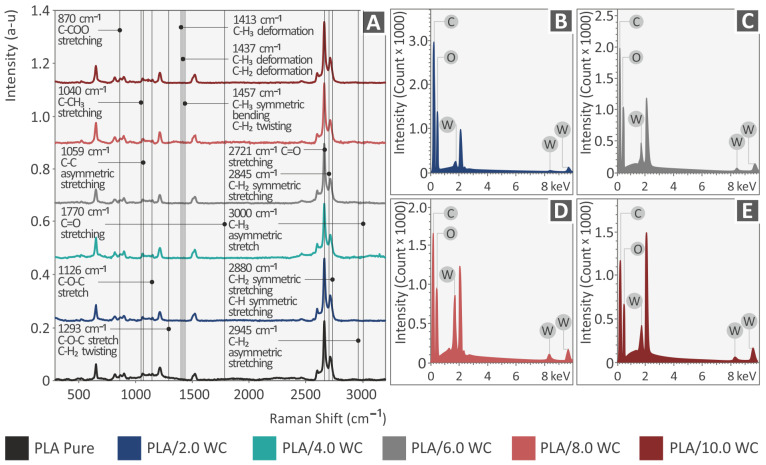
(**A**) Raman spectra from pure PLA, PLA/2.0 WC, PLA/4.0 WC, PLA/6.0 WC, PLA/8.0 WC, and PLA/10.0 WC and EDS investigation for the PLA/WC composites: (**B**) PLA/2.0 WC, (**C**) PLA/6.0 WC, (**D**) PLA/8.0 WC, and (**E**) PLA/10.0 WC.

In Figure 6, it can be observed that the Raman spectra derive mainly from the pure PLA material. Except for two gradual increases that are near the noise level, there are no discernible Raman spectrum changes caused by the WC additive. The rest of the changes observed have a random behavior, indicating material inhomogeneity rather than the presence of a WC additive. Particularly, the initial difference is an escalation at 2848 cm^−1^ (C-H_2_ symmetric stretching) and the second is an increase at 2883 cm^−1^ (C-H_2_ or C-H symmetric stretching). All differences show a gradual increase in the percentage of the WC additives used. The associated Raman peaks from the pure PLA specimen are shown in Table 2 and are supported by the corresponding literature.

### 3.4. Evaluation of the Filament Performance

**Figure 7:**
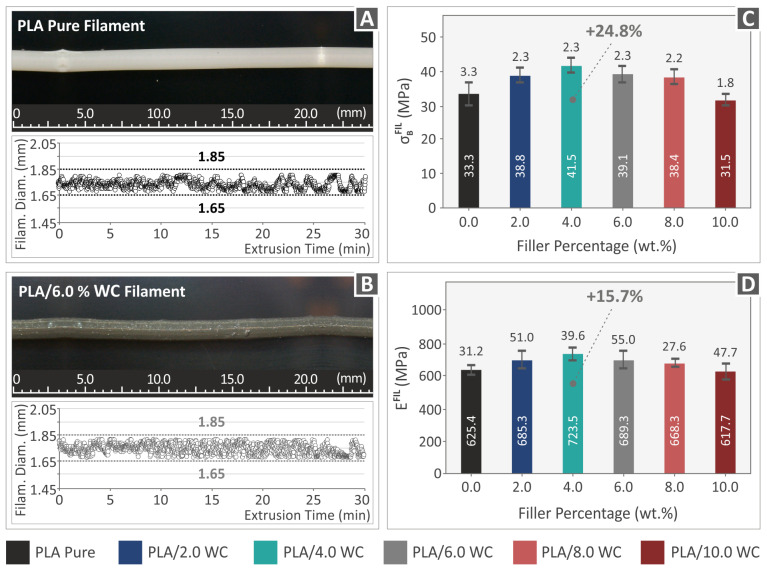
Real-time measurements of the extruded filament segments for two types of filaments: (**A**) PLA and (**B**) PLA/6.0 WC. (**C**) The results of the tensile experiments performed on the filament and (**D**) the obtained results for the filament’s modulus of elasticity.

As previously noted, the investigated filaments were produced employing a composer extrusion device by 3devo BV in Utrecht, Netherlands, to ensure a consistent filament quality. A closed-loop system was installed in the machine to control the filament diameter during the extrusion procedure. This integrated system was used to construct a consistently sized filament accompanied by precise diameter measurements. Adjustments were made as needed within the allowable device limits. Figure 7A,B illustrate two portions of the created filaments selected at random. The snapshots were obtained utilizing an OZR5 optical stereoscope manufactured by KERN & SOHN GmbH in Albstadt, Germany. The images show that the pure PLA filament (Figure 7A) displays a smooth surface without any noticeable defects. Conversely, the PLA/WC 6 wt. % filament (Figure 7B) exhibits a generally smooth surface but with small protrusions present. The figures also present the dynamic monitoring of the filament diameter for both the pure PLA and PLA/WC 6 wt. % composites. The data analysis reveals a minor variation of approximately 200 μm in the filament diameter measurements, which falls within an acceptable range for MEX 3D printing. These findings validate the suitability of the investigational approach and validate the appropriate selection of the control variables.

The experimental outcomes of the filament’s tensile strength are shown in Figure 7C. The results indicate a notable increase in tensile strength for all WC percentages, with the exception of the PLA/WC 10.0 wt. % filament, which showed a minor reduction in comparison to the raw PLA filament. Additionally, compared to the raw PLA filament, the PLA/WC 4.0 wt. % filament showed the greatest improvement in tensile strength, which had an increase of up to 24.8%. The influence of the WC nanoparticles on the stiffness of the generated filaments is seen in Figure 7D. It is worth mentioning that the filament stiffness was also altered by the inclusion of WC particles, with an increase of up to 15.7% in stiffness when compared to the pure PLA filament, as reported for the PLA/WC 4 wt. % filament (Figure 7D).

**Figure 8:**
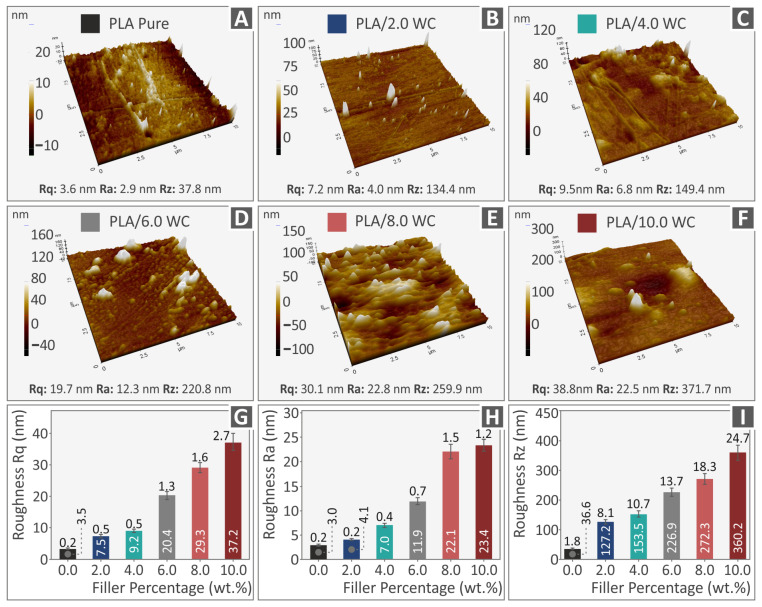
The images captured from the lateral surfaces of the investigated filaments using AFM are displayed for various compositions: (**A**) pure PLA, (**B**) PLA/2.0 WC, (**C**) PLA/4.0 WC, (**D**) PLA/6.0 WC, (**E**) PLA/8.0 WC, and (**F**) PLA/10.0 WC. Graphs depicting the relationship between the WC concentration and surface roughness parameters in the compounds are presented for (**G**) Rq, (**H**) Ra, and (**I**) Rz.

All of the generated filaments’ lateral surfaces underwent an AFM (Atomic Force Microscopy) inspection. The findings of this examination are presented in Figure 8, which demonstrates that all of the filaments had rougher surface textures than those of the pure PLA ones. The strong impact of adding WC to the PLA matrix on the filament morphology is amply demonstrated by the observed linear and progressive increase in surface roughness values, which is correlated with the increase in WC concentration. Rq (root-mean-square roughness), Ra (average of surface heights and depths across the surface), and Rz (difference between the tallest “peak” and the deepest “valley” in the surface), i.e., three surface roughness parameters, vs. the amount of WC in the composites are plotted in Figure 8G–I to demonstrate their relation. As shown, an increase in the WC content results in higher values for each of the three surface roughness measurements. It is noteworthy that the ability of the filament to be processed during the 3D-printing procedure could be affected as a result of this alteration of the filament morphology, which also denotes a decline in the final filament quality [82].

### 3.5. Evaluation of the 3D-Printed Specimens’ Mechanical Properties

**Figure 9:**
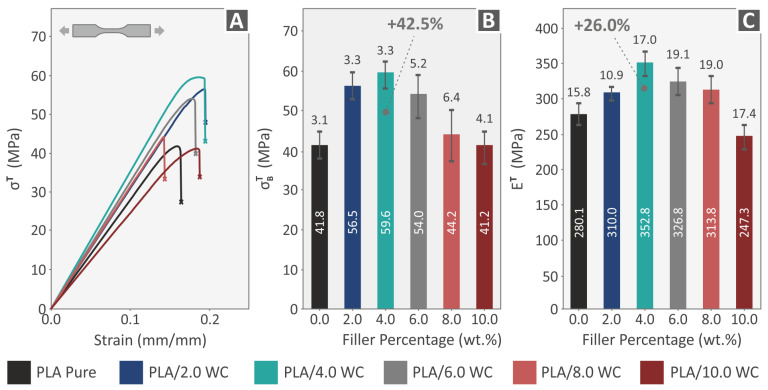
The outcomes derived from the tensile testing of the 3D-printed specimens: (**A**) representative graphs representing the correlation of tensile stress and strain for one 3D-printed sample selected at random from each nanocomposite material, (**B**) mean values of the tensile strength, and (**C**) mean values of the tensile modulus of elasticity.

The experimental examination for the 3D-printed specimens was conducted in line with ASTM D638-02a guidelines. Graphs showing the linkage between the estimated strain (mm/mm) and tensile stresses (MPa) of a randomly selected sample of each composite material as well as the pure PLA are shown in Figure 9A. The average tensile strength and deviation from the five specimens tested vs. the filler percentage is represented in Figure 9B. In Figure 9C, the tensile modulus of elasticity for the pure PLA and each loading percentage are also displayed. According to the test findings, all samples with various WC concentrations have superior tensile properties than the pure PLA ones. The sample with 4.0 weight percent WC outperformed the others, showing a noticeable improvement in stiffness (26.0%) and tensile strength (42.5%). The findings indicate that the compounds’ ability to improve the mechanical characteristic of the composite has a physical limit, with the maximum values being attained at a weight concentration of 4.0 percent. The compounds at increasing loadings come close to the WC additive’s saturation threshold in the thermoplastic PLA, as seen by the diminishing mechanical characteristics with loadings greater than 4.0%.

**Figure 10:**
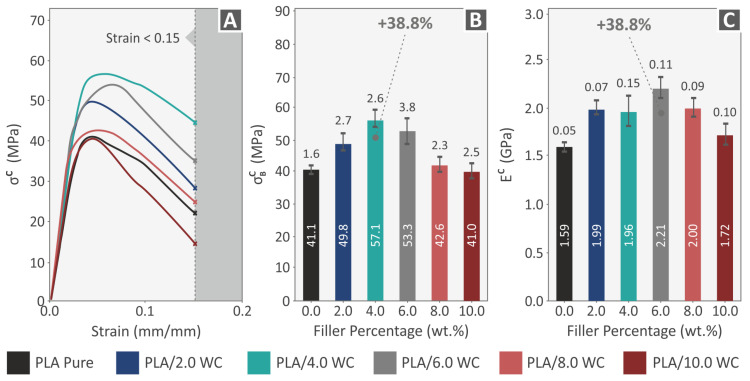
The series of compression tests to which all specimens were subjected. (**A**) Compression stress versus calculated strain graphs for a specimen chosen at random from each nanocomposite, representing one of the five printed specimens; (**B**) standard deviations and average results for compression strength; and (**C**) mean results of the compression modulus of elasticity.

According to the ASTM D695 standard, Figure 10 presents a summary of the compression experiments conducted on the produced specimens consisting of pure PLA and PLA/WC composites. The experiments were terminated at 15% strain, as it was considered that no valuable information could be derived from the experimental process beyond this point. Figure 10A shows the examined specimen’s compression stress–strain graphs and Figure 10B shows the corresponding average compression strength. The 3D-printed samples’ typical compression modulus of elasticity is shown in Figure 10C. The analysis of the compression experimental data revealed that the compression strength (Figure 10B) followed a pattern that was comparable to the tensile strength. Except for the composite with a filler concentration of 10.0 wt. %, all the other composites demonstrated an increase in compression strength. Similar to what was observed in the tensile experiments, there was a threshold at which the compression strength reached its greatest value at a concentration of 4.0 wt. % WC, which was 38.8% in comparison to that of the raw PLA. Additionally, the compound containing 6 wt. % of WC displayed the greatest improvement in the compression modulus of elasticity (Figure 10C), showing a remarkable increase of 38.8% over the pure PLA.

**Figure 11:**
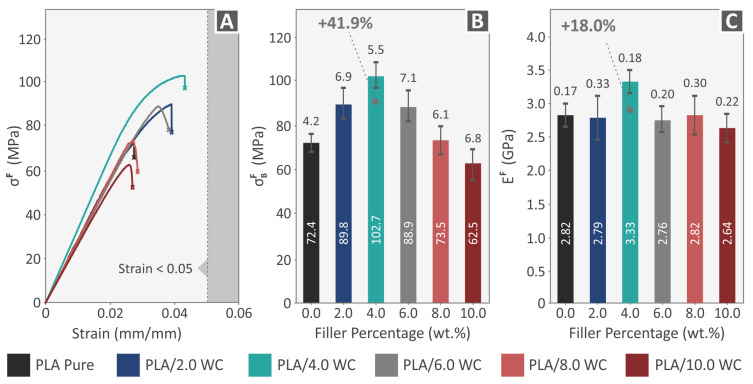
The results of the flexural testing performed on all 3D-printed specimens. (**A**) Stress–strain curves for diverse specimens evaluated for flexural strength, with one specimen chosen at random from each nanocompound; (**B**) mean results and deviations of the flexural strength findings; and (**C**) mean results and deviations of the flexural modulus of elasticity results.

A review of the flexural characteristics of the samples made from the pure PLA and PLA/WC composites is presented in Figure 11. Figure 11A exhibits the flexural stress–strain curves. The 3D-printed samples’ typical values of the flexural strength and modulus of elasticity are shown in Figure 11B,C, correspondingly. The ASTM D790-10 standard instructs that the flexural examinations should be terminated at a maximum strain of 5%. The samples tested in this paper failed at lower strain values; so, the 5% threshold instructed by the standard was not reached. All the composites showed noticeable gains in their flexural strength, with the exception of the samples with the greatest WC filler loading (10.0 wt. %). The greatest improvement in flexural strength occurred at a compound loading of 4.0 wt. %, which resulted in maximum flexural strength of 102.7 MPa, with a considerable increase of 41.9% over that of the raw PLA. The compound with a 4.0 wt. % loading also showed the maximum flexural modulus of elasticity with a value of 3.33 GPa, demonstrating a considerable increase of 18.0% in relation to the raw PLA sample. It can be assumed that the improvement in the flexural characteristics of the specimens reached the saturation point after the 4.0 wt. % WC filler loading.

**Figure 12:**
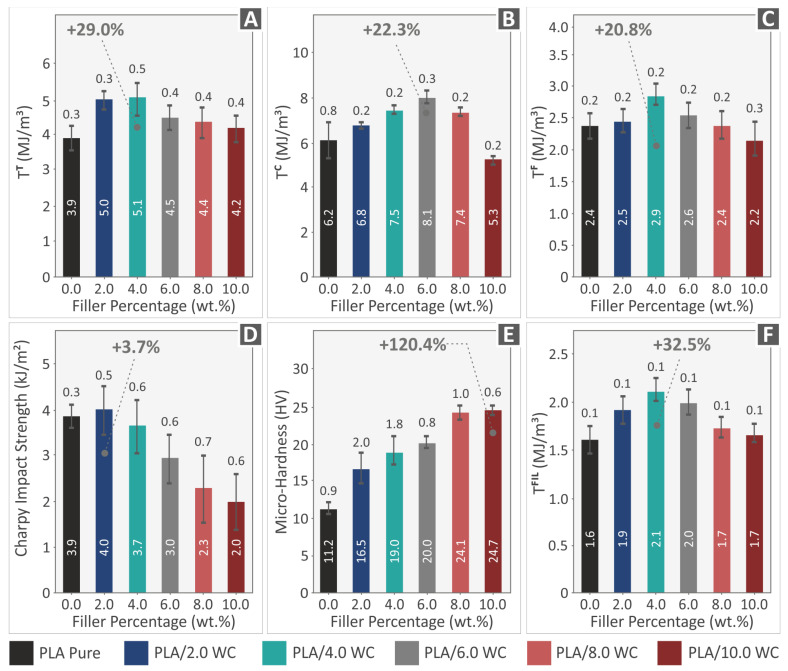
The average values and standard deviations of the mechanical properties for all fabricated specimens and filaments. (**A**) Tensile toughness of the samples, (**B**) compression toughness of the samples, and (**C**) flexural toughness of the constructed samples. (**D**) Impact strength, (**E**) Vickers microhardness values, and (**F**) filaments’ tensile toughness.

The toughness measurements that were obtained from the mechanical tests, both in filament and specimen forms, are shown in Figure 12A–C,F. Stress–strain graphs were employed to determine the amount of energy absorbed throughout testing. Through the integration of the associated stress vs. strain curves, the toughness values for tensile, compression, and flexural testing were obtained. Knowing a material’s toughness values (the energy the material absorbs in each experiment) helps to determine its fracture characteristics and allows for the incorporation of a “safe-fail” procedure for a variety of applications. Figure 12A–C,F show that the composites display higher values of tensile, compression, and flexural toughness than the pure PLA polymer, with the exception of the specimens with 10.0 wt. % loading, which showed a modest decrease in their compression and flexural toughness, owing to the inferior performance of the nanocomposites in these tests.

Figure 12D,E reveal the results of the impact experiments and Vickers microhardness assessments, respectively. The mean Charpy impact strength (in kJ/m^2^) and Vickers microhardness (in HV) were evaluated for each material studied with respect to the filler concentration. The impact behavior of the materials exhibited distinct patterns. Only the specimens with a concentration of 2.0 wt. % WC demonstrated an improvement in the impact strength, with only a marginal 3.7% increase (in the statistical error order). In contrast, all other specimens with filler concentrations ranging from 4.0 to 10.0 wt. % exhibited a linear decrease in their impact strength compared to the pure PLA ones. The results of the Vickers microhardness tests demonstrated a consistent and significant increase in hardness across all the tested composites. Particularly, the composites with a 10.0 wt. % WC loading exhibited the highest improvement in microhardness, showing a value of 24.7 HV and a remarkable 120.4% increase when compared to the pure PLA material. This indicates that the incorporation of WC filler particles effectively enhances the overall hardness and strength of the specimens, making them well-suited for applications that require resistance to wear, scratching, and localized stresses.

### 3.6. Three-Dimensionally Printed Specimens’ Morphological Analysis

**Figure 13:**
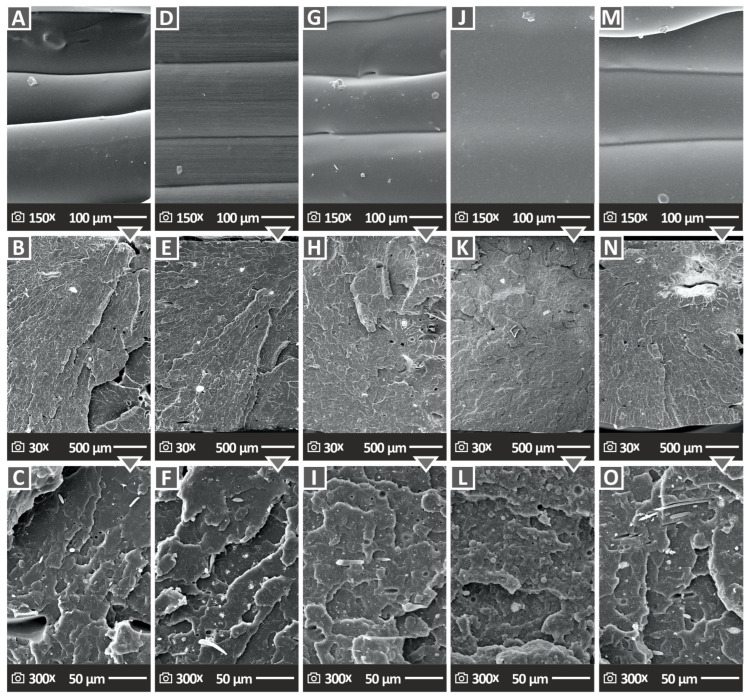
SEM images illustrating the surfaces of the specimens at different magnifications. (**A**) Lateral surface of PLA/WC 2 wt. % at 150× enlargement, (**B**) cracked surface of PLA/WC 2 wt. % at 30× enlargement, (**C**) cracked surface of PLA/WC 2 wt. % at 300× enlargement, (**D**) lateral surface of PLA/WC 4 wt. % at 150× enlargement, (**E**) cracked surface of PLA/WC 4 wt. % at 30× enlargement, (**F**) cracked surface of PLA/WC 4 wt. % at 300× enlargement, (**G**) lateral surface of PLA/WC 6 wt. % at 150× enlargement, (**H**) cracked surface of PLA/WC 6 wt. % at 30× enlargement, (**I**) cracked surface of PLA/WC 6 wt. % at 300× enlargement, (**J**) lateral surface of PLA/WC 8 wt. % at 150× enlargement, (**K**) cracked surface of PLA/WC 8 wt. % at 30× enlargement, (**L**) cracked surface of PLA/WC 8 wt. % at 300× enlargement, (**M**) lateral surface of PLA/WC 10 wt. % at 150× enlargement, (**N**) cracked surface of PLA/WC 10 wt. % at 30× enlargement, and (**O**) cracked surface of PLA/WC 10 wt. % at 300× enlargement.

The fractured and lateral surfaces of the 3D-printed samples in the tensile test were inspected thoroughly with SEM imaging, which also allowed for detailed observation and investigation at high magnifications. Five samples are shown in Figure 13A,D,G,J,M with their lateral aspects magnified (at 150×). These samples are PLA/WC 2 wt. %, PLA/WC 4 wt. %, PLA/WC 6 wt. %, PLA/WC 8 wt. %, and PLA/WC 10 wt. %, respectively. Except for the sample with a 6.0 wt. % filler loading, which shows slight voids, the interconnections among the layers appear to be defect-free and without voids based on the overall observation and investigation of the samples. It is worth mentioning that the samples do not exhibit consistent layer shapes and, in the case of the 8.0 wt. % filler loading, the interconnections between layers are not even distinguishable.

Figure 13B,E,H,K,N depict the fractured surface at a 30× magnification, and Figure 13C,F,I,L,O depict the fractured surface at a 300× magnification. All of the specimens as shown at 30× magnification exhibited microvoids, which are attributed to the layer-by-layer construction of the samples during the MEX 3D-printing process, even though a 100% infill ratio was used. It is worth mentioning that these microvoids are frequently seen in the structure of 3D-printed objects [83]. The higher magnification of 300× of the samples using SEM imaging demonstrated that the filament strands were not significantly deformed.

According to the SEM sample examination, the samples appear to have absorbed moisture since there are microvoids and microporosities present [84,85]. It is crucial to emphasize this observation because the microvoids are not located within the filament itself and, as a result, they do not impact the specimen’s total effectiveness. The compounds had greater degrees of deformation, which suggests that the samples’ fracture mechanisms were more ductile. This implies that the materials possess a higher capacity for plastic deformation prior to fracture.

**Figure 14:**
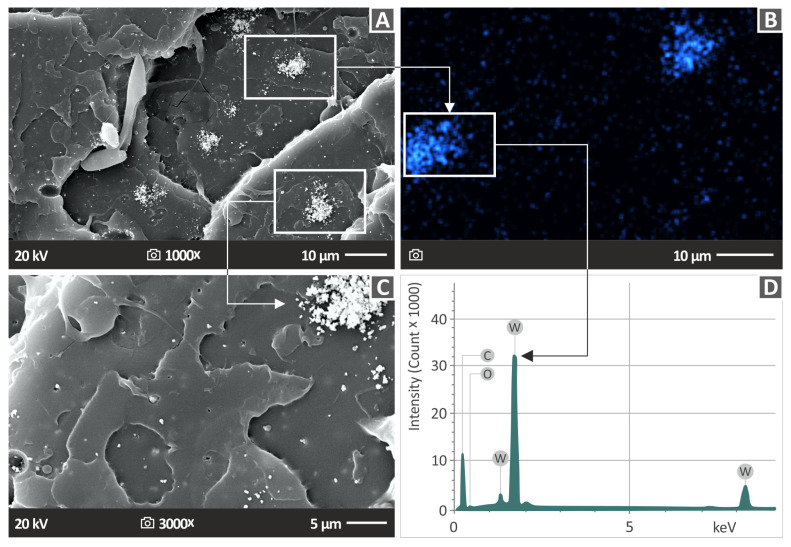
SEM images illustrating: (**A**) PLA/WC 10.0% at 1000× enlargement, (**B**) tungsten EDS map, (**C**) PLA/WC 10.0 wt. % at 3000× enlargement, and (**D**) EDS assessment for PLA/WC 10.0 wt. % obtained from a region with an abundance of WC particles.

Figure 14 shows the filler dispersion within the PLA polymer and assesses the presence of any potential agglomerations. A 1000× magnification SEM image of the PLA/WC 10% composite is shown in Figure 14A, demonstrating the presence of clustered WC particles. Microporosity on the surface and clustered WC particles can be seen in Figure 14C, which shows a 3000× magnification of the region displayed in Figure 14A. Additionally, Figure 14B shows an EDS map of the region seen in Figure 14A, specifically for the W element. The EDS mapping’s distribution of the W element provides evidence that the particles seen in Figure 14A are, in fact, WC particles. A location with a higher concentration of W denotes an agglomeration of the particles. The analogous EDS graph from the agglomeration area in the specimen with a 10 wt. % filler loading is shown in Figure 14D. Tungsten has a noticeable peak on the EDS graph, showing a large volume of this element concentrated in the region under examination. These phenomena refer to the nanoparticles’ mechanism and are expected to occur to some extent in all WC loadings. As the filler loading increases, the nanoparticles cover all the gaps between the polymeric chains and their excess produces clusters, such as the ones shown in Figure 14. The are many studies and theories about the formations of such clusters and their effect on the mechanical and overall performance of nanocomposites [86,87].

## 4. Discussion

WC is rarely employed as a reinforcement in thermoplastics, as shown in the literature review presented in this study. Therefore, it was a challenging and interesting issue to address, covering this specific gap in the literature. Whether the superior properties of the WC ceramic can be induced in nanocomposites was also a main issue to be answered in the study. The literature suggests that each filler has a different effect on each matrix; so, the outcome cannot be predicted. Each material is suitable for specific types of applications. The corresponding nanocomposites containing these materials can improve the performance in applications in which these materials are already used. For example, WC is used in coatings due to its resistance to wear. The findings reported in this paper confirmed these capabilities, as the nanocomposites showed increased microhardness, which is an indication of good resistance to wear for the specific nanocomposites. Other fillers, such as glass fibers [88], would have affected the PLA matrix differently and would have produced composites for different types of applications. Additionally, the particle size affects the reinforcing effect of the filler [89]. So, the overall response of the composites differs regarding not only the mechanical reinforcement but also the thermal, structural, and rheological properties. Such differences affect the processability of the composites, especially in the 3D-printing process, and justify the need for such types of studies for each different matrix and filler combination.

**Figure 15:**
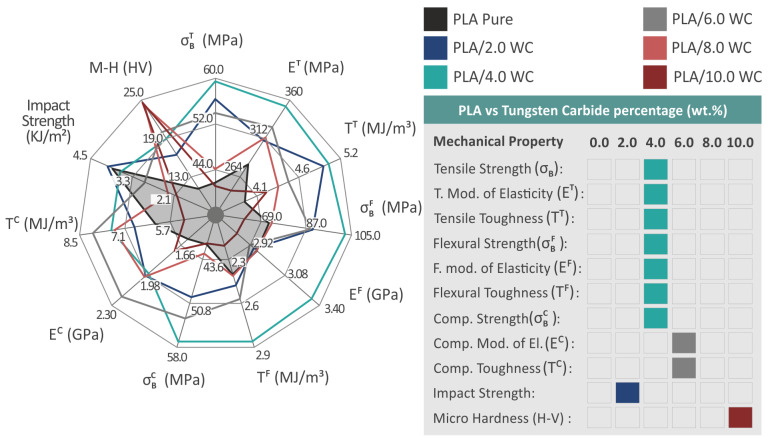
The mechanical behavior of the specimens created via 3D printing is shown in the spider chart on the left. The performance of pure PLA is shown in the gray area, which was utilized as a reference point for reviewing the successes of improvements. The materials that showed superior mechanical performance in all trials are displayed in the index on the right.

A summary of the mechanical experiments conducted on the composites subjected to examination and the raw PLA material is illustrated in Figure 15. The introduction of WC NPs into the PLA matrix enhanced the polymer’s mechanical response.

The 4.0 weight percent compound in the assessments was shown to have the greatest mechanical properties in most of the tests. Therefore, it can be established that, among the created and evaluated compounds, this loading is the best option. The PLA/WC 4 weight percent specimens in particular displayed the ultimate enhancement in tensile and flexural properties, as well as a considerable improvement in compression strength. It is significant to mention that the greatest results seen at the 4.0 wt. % loading in the majority of the mechanical tests, as mentioned before, indicates that the improvement in the mechanical characteristics is directly related to the concentration of WC up to this point. The saturation of WC in the PLA matrix takes place at higher concentrations, as evidenced by the fact that increasing the WC content above the 4 wt. % threshold results in a decrease in the mechanical characteristics. Since it was beyond the scope of the research, the precise saturation threshold was not determined in the present study. According to the nanoparticles’ mechanism, the increase in the filler concentration in the matrix leads to the formation of a network between the polymeric chains making them stiffer, which ultimately leads to increased mechanical properties. After a specific filler loading, a saturation of the filler in the matrix occurs, which leads to a decrease in the mechanical response. The filler percentage in the matrix at which this starts to occur depends on various parameters, and it is not an obvious and constant percentage. It needs further investigation to be determined for each filler–matrix combination [86,87,90,91,92].

Furthermore, the compression toughness and elastic compression modulus both noticeably increased up to a 6.0 weight percent filler loading, for which the highest achieved values of 38.8% and 22.3% were observed, respectively. The microhardness showed a steadily rising improvement, reaching a maximum of 120.4% at an additive loading of 10 wt. % when compared to the pure matrix. Such a high performance in the microhardness tests was expected due to the specification of the WC ceramic and makes the nanocomposites investigated in this paper suitable for applications requiring high wear resistance from the material. Still, it should be noted that the performance of the 10 wt. % nanocomposite in the remaining mechanical tests was not as high. Therefore, according to the specifications set in each application, the WC concentration in the nanocomposite should be adjusted accordingly. A high strength in the tensile and flexural tests can be achieved with 4 wt. % loading and, in cases in which high wear is the priority, WC can be further increased up to 10 wt. %. Only the impact strength exhibited a reduction in the final mechanical responsiveness. All the compounds showed decreased values than those of the PLA material, with the exception of the PLA/WC 2.0 wt. %, which showed an increase when compared to the pure PLA. Except for their performance under impact loads, the improvement in the generated compounds appears to be directly influenced by the WC content. The decrease in the mechanical performance of the nanocomposite with the highest loading of 10 wt. % can be attributed to the saturation of the WC nanoparticles in the nanocomposites. In this work, the filler loading was gradually increased, the mechanical tests were conducted, and the experiments stopped when the mechanical performance started to consistently decrease. The saturation of the filler has a negative effect on the mechanical response of the nanocomposites [93], and this was the outcome in most of the mechanical properties except for the microhardness, which was increased. At the 10 wt. % loading, agglomerations of the WC particles were also formed, which also negatively affects the mechanical response [94]. The increase in the microhardness with the increase in the filler was expected. The increased concentration of the very-hard WC nanoparticles in the nanocomposites led to this effect.

The microstructures of the 3D-printed object’s lateral surface can be analyzed to gain valuable information about key features, including the layer’s thickness, the fusion of the molten polymer, the interfaces among the layers, and the general caliber of the 3D-printing process. According to the SEM images, the presence of microvoids on the 3D-printed specimen’s surface is not increased by the rise in the filler concentration within the PLA matrix. It is important to highlight that the samples did not exhibit homogeneous layer morphologies in the case of the 8.0 wt. % filler loading. The connections among the layers were not easily discernible. As was previously noted, the discovered flaws in the structural integrity of the samples were to be expected during the MEX 3D-printing process.

A low MFR means that a high viscosity leads to structures with increased porosity, due to the reduction in the material flow. This has an effect on both the 3D-printed structure and the mechanical performance of the samples, as it was shown by correlating the rheological properties with the mechanical test results. A higher MFR (lower viscosity) leads to a more solid construction. To improve this issue, the 3D-printing settings need to be optimized for each nanocomposite, as a low MFR requires at least higher temperatures to achieve the same material flow. In this study, the 3D-printing settings were optimized for the unfilled thermoplastic PLA and the same settings were also used for the nanocomposites to have comparable results (samples produced with the same conditions). 

To quantify the porosity and microporosity of the 3D-printed structure, a sophisticated method, such as micro-computed tomography (μ-CT), is required. Such an investigation was not within the scope of the work, in which a laborious effort with fifteen different tests was conducted to characterize the produced nanocomposites. The main aim was to evaluate the performance of WC nanoparticles as reinforcing agents in the most popular polymer in MEX 3D printing, that is, the PLA polymer. Studying the internal structure of the samples and how it is affected by the filler loading can be the subject of future work. On the other hand, making estimations regarding the size and the number of microvoids in the 3D-printed structure by inspecting the SEM images does not provide reliable results. SEM images show only one cross-section of the samples, which is additionally deformed by the failure of the sample in the mechanical tests. The failure is expected to affect both the number and the size of such formations. Furthermore, 2D SEM images, although accurate and with scale bars, are not an adequate means to measure 3D structures, such as the porosity and voids in the 3D-printed structure. It is significant to emphasize that the TGA research verified that the employed temperatures in the MEX procedure have no negative impact on the materials used. This evaluation is important because it guarantees that temperature-induced damage will not harm the 3D-printing procedure itself or the mechanical behavior of the specimens created when using the manufactured composites.

Tungsten carbide (WC) has been used as a reinforcing material in different polymeric matrices for MEX 3D printing. In one study, composites of PTFE filled with tungsten carbide (WC) in weight percentages of 5.0%, 10.0%, 20.0%, and 30.0% were examined. The sample of the composites with a 20% WC filler was distinguished from the others in terms of tribological and mechanical characteristics [95]. In an additional investigation, the measurements of the tensile strength and percentage of elongation in laminated constructions were taken using a computerized universal testing apparatus. The basalt fiber-reinforced laminate with 6.0% tungsten carbide filler outperformed the other laminate combinations in terms of strength during testing. The addition of tungsten carbide fillers showed a synergistic impact in enhancing tensile characteristics and the percentage of elongation in epoxy composites [96]. The reinforcement achieved in this paper in the thermoplastic PLA by the addition of WC NPs was similar or higher to that reported in the literature. Comparing the findings presented in this paper with the corresponding results from the literature for the ABS polymer in MEX 3D printing, with nanocomposites prepared using a similar process to that of the present study, it was found that the reinforcing effect of WC nanoparticles on the PLA polymer was significantly higher in all the mechanical tests conducted. More specifically, a 42.5% increase in the tensile strength is reported in this paper, while the corresponding value for the ABS polymer was 29.4%. In the compression strength, a 38.8% increase was achieved in this paper, compared to a 25.9% increase in the ABS polymer. In the flexural strength, there was a 41.9% increase for the PLA polymer vs. 20.9% for the ABS polymer. Finally, the microhardness of the PLA polymer was increased by 120.4%, while it was increased by 100.3% for the ABS polymer. The ABS polymer surpasses the PLA polymer only in the increase in its stiffness on the tensile and flexural tests. On the compression test, the modulus of elasticity of the PLA polymer was increased by 38.8% compared to 20.4% on the ABS polymer [55].

## 5. Conclusions

This study investigated a novel use of ceramics, such as tungsten carbide (WC), as a strengthening factor for PLA materials used in MEX 3D printing, revealing also its advantages. The study’s main goal was to determine whether adding WC particles improves the mechanical properties of the PLA polymer. The experiments effectively revealed that the addition of WC ceramic powder to the PLA matrix can significantly increase the mechanical performance of the thermoplastic PLA polymer. Numerous tests were executed using 3D-printed specimens to fully examine the mechanical behavior of the produced composite polymer when combined with WC particles.

Various mechanical properties were significantly improved as a result of the incorporation of WC ceramic particles into the PLA polymer matrix. Tensile strength, compression strength, and flexural strength all significantly improved when compared to pure PLA, with improvements of 42.5%, 38.8%, and 41.9%, respectively. Additionally, increases of 20.8%, 22.3%, and 29.0% were found in flexural toughness, compression toughness, and tensile toughness, respectively. Furthermore, there were improvements in the tensile, compression, and flexural modulus of elasticity, measuring 26.0%, 38.8%, and 18.0%, respectively. Mechanical testing was conducted in accordance with international standards. Due to the hardness of the ceramic additive, the addition of WC to the composites also resulted in a considerable improvement in the microhardness, with a maximum improvement of 120.4%, making the nanocomposites compatible with specialized corresponding applications.

The outcomes of this paper encourage the fabrication of composite materials with better mechanical properties for the MEX 3D-printing method. Exploring the procedures necessary for industrialization, such as enlarging the production process and pinpointing the precise percolation threshold of the WC filler in the polymeric material, can be topics of future research. The strengthening impact of the WC filler in the PLA polymer can also be improved by adjusting the 3D-printing parameters and, thus, maximizing the advantages of the composite material. By addressing these aspects, the practical value of the study’s findings can be further increased, facilitating the adoption of WC-reinforced PLA composites in various industries.

## Figures and Tables

**Table 1:** Temperature and enthalpy values of the characteristic transitions.

	Tg (°C)	Tc (°C)	Tm (°C)	ΔHc (J/g)	ΔHm (J/g)	Xc (%)
PLA Pure	59.47	89.37	150.68	−11.117	30.473	20.81
PLA/2.0 WC	59.57	120.47	151.82	−6.9015	24.608	21.24
PLA/4.0 WC	58.89	121.91	151.92	−5.8795	21.054	21.68
PLA/6.0 WC	60.15	123.55	152.41	−3.5875	15.599	22.14
PLA/8.0 WC	59.91	122.39	152.06	−5.0402	21.534	22.62
PLA/10.0 WC	59.83	123.21	151.83	−7.4067	19.716	23.13

**Table 2:** The determination of the major Raman peaks in the pure PLA and their assigned values.

Wave Number (cm^−1^)	Intensity	Raman Peak Assignment
870	Medium	C-COO stretching [75]
1040	Small	C-CH3 stretching [75]
1059	Small	C−C asymmetric stretching
1126	Medium	C-O-C stretching [76]
1293	Medium	C-O-C stretching [76]; C-H_2_ twisting [77]
1413	Small	C-H_3_ deformation [78]
1437	Medium	C-H_3_ deformation [78] C-H_2_ deformation [77]
1457	Medium	C-H_3_ symmetric bending [75,76]; C-H_2_ twisting [77]
1770	Medium	C=O stretching [75,76]
2721	Small	C=O stretching [79]
2845	Major	C-H_2_ symmetric stretching [80]
2880	Major	C-H_2_ symmetric stretching [80]; C-H symmetric stretching [81]
2945	Major	C-H_2_ asymmetric stretching [80]
3000	Medium	C-H_3_ asymmetric stretch [81]

## Data Availability

Data are available upon request.
